# Sterile Cerebrospinal Fluid Culture at Cryptococcal Meningitis Diagnosis Is Associated with High Mortality

**DOI:** 10.3390/jof9010046

**Published:** 2022-12-28

**Authors:** Caleb P Skipper, Katherine Huppler Hullsiek, Anna Stadelman, Darlisha A Williams, Kenneth Ssebambulidde, Elizabeth Okafor, Lillian Tugume, Edwin Nuwagira, Andrew Akampurira, Abdu K Musubire, Mahsa Abassi, Conrad Muzoora, Joshua Rhein, David R Boulware, David B Meya

**Affiliations:** 1 Department of Medicine, University of Minnesota, Minneapolis, MN 55455, USA; 2 Infectious Diseases Institute, Makerere University, Kampala 7062, Uganda; 3 School of Public Health, University of Minnesota, Minneapolis, MN 55455, USA; 4 Department of Medicine, Mbarara University of Science and Technology, Mbarara 1410, Uganda

**Keywords:** Cryptococcus, HIV, AIDS, cryptococcal meningitis

## Abstract

Cryptococcus is the leading cause of AIDS-related meningitis in sub-Saharan Africa. The clinical implications of a sterile cerebrospinal fluid (CSF) culture among individuals diagnosed with cryptococcal meningitis using CSF cryptococcal antigen (CrAg) are unclear. We prospectively enrolled 765 HIV-positive Ugandans with first-episode cryptococcal meningitis from November 2010 to May 2017. All persons were treated with amphotericin-based induction therapy. We grouped participants by tertile of baseline CSF quantitative Cryptococcus culture burden and compared clinical characteristics, CSF immune profiles, and 18-week mortality. We found 55 (7%) CSF CrAg-positive participants with sterile CSF cultures. Compared to the non-sterile groups, participants with sterile CSF cultures had higher CD4 counts, lower CSF opening pressures, and were more frequently receiving ART. By 18 weeks, 47% [26/55] died in the sterile culture group versus 35% [83/235] in the low culture tertile, 46% [107/234] in the middle tertile, and 56% [135/241] in the high tertile (*p* < 0.001). The sterile group had higher levels of CSF interferon-gamma (IFN-γ), IFN-α, interleukin (IL)-6, IL-17, G-CSF, GM-CSF, and chemokine CXCL2 compared with non-sterile groups. Despite persons with sterile CSF cultures having higher CD4 counts, lower CSF opening pressures, and CSF cytokine profiles associated with better Cryptococcus control (e.g., IFN-γ predominant), mortality was similar to those with higher fungal burdens. This unexpected finding challenges the traditional paradigm that increasing CSF fungal burdens are associated with increased mortality but is consistent with a damage-response framework model.

## 1. Introduction

Cryptococcus neoformans is the leading cause of AIDS-related meningitis in sub-Saharan Africa [1,2,3]. Cryptococcal meningitis is often diagnosed by detecting cryptococcal antigen (CrAg) in cerebrospinal fluid (CSF), although CSF fungal culture is the traditional gold standard. CrAg testing has proven more sensitive than fungal culture in the CSF [4]; 10–15% of persons with symptomatic cryptococcal meningitis may be CrAg positive yet without any Cryptococcus yeast isolated on culture. In ART-naïve populations, higher CSF fungal burdens generally correlate with increasing mortality in first-episode cryptococcal meningitis [5]. However, it is unknown whether persons with no culture growth (i.e., sterile CSF) have lower mortality per the traditional paradigm.

Despite increasing access to ART throughout Africa in the last decade, many persons still present with advanced HIV disease [6]. In general, routine HIV care has moved away from CD4 testing at diagnosis and rather focused on early ART initiation and viral load monitoring. Test-and-treat strategies may carry a disadvantage in persons with early cryptococcal disease, as initiating ART without antifungal therapy could unmask cryptococcal meningitis. Rhein et al. demonstrated that persons who developed cryptococcal meningitis after initiating ART within the prior 14 days of diagnosis had 2-fold worse mortality [7]. It is becoming increasingly common to encounter ART-experienced persons with cryptococcal meningitis, but it remains unknown what impact ART might have on those with cryptococcal meningitis but sterile CSF cultures.

During a large randomized trial [8], we found a notable proportion of persons with cryptococcal meningitis who presented with positive CSF CrAg LFA but with sterile CSF fungal cultures [9]. In order to study this unique patient subset, we subsequently identified all persons in our database with sterile CSF cultures over a 7-year period of prospective enrollment. We compared the baseline characteristics, CSF cytokine profiles, and 18-week survival outcomes of persons with sterile CSF against those in tertile groupings of low, middle, or high CSF quantitative Cryptococcus growth, and analyzed the impact that recently initiated ART might play in this unique subset of persons with cryptococcal meningitis.

## 2. Materials and Methods

We prospectively enrolled HIV-positive persons with cryptococcal meningitis into clinical cohorts from November 2010 to May 2017 in Kampala and Mbarara, Uganda. We screened hospitalized persons with clinical evidence of meningitis by lumber puncture for cryptococcal meningitis using the CrAg lateral flow assay (LFA) (IMMY, Norman, Oklahoma, USA). Participants would then typically enter either of the two main trials occurring over that period. The Cryptococcal Optimal ART Timing (COAT) trial and the Adjunctive Sertraline for the Treatment of HIV-Associated Cryptococcal Meningitis (ASTRO-CM) trials have been previously described [8,10,11]. Full inclusion and exclusion criteria can be found at ClinicalTrials.gov (NCT01075152; NCT01802385). All participants received antifungal induction therapy at meningitis diagnosis with amphotericin B deoxycholate (0.7–1.0 mg/kg/day) and fluconazole (800 mg/day) for up to 2 weeks, followed by fluconazole consolidation and maintenance therapy. All participants also received key supportive therapies including therapeutic lumbar pressures for elevated intracranial pressure, intravenous fluids, and electrolyte repletion. The COAT trial followed participants for 46 weeks, while the primary endpoint for the ASTRO-CM trial was 18-week survival. This analysis includes participants enrolled in any of the dose-finding pilot studies, the main interventional trials, or an open label compassionate care arm over that time period.

Quantitative CSF cultures were obtained on all participants at baseline, day 3, 7, and 14, as described [12]. Cryptococcal meningitis diagnosis was based on a positive CrAg LFA in the CSF regardless of CSF culture positivity. Quantitative CSF cultures were performed with five serial 1:10 dilutions of 100 μL of CSF. Sterile cultures were defined as no growth of Cryptococcus after 10 days of incubation on Sabouraud Dextrose agar (1 colony forming unit (CFU)/mL limit-of-detection) [12]. Clinical characteristics, laboratory results, and outcomes were collected via study-specific case report forms through the 18 weeks of study follow up.

To assess whether persons with sterile CSF cultures had an immune response differing from those with fungal growth, we measured biomarkers from cryopreserved baseline CSF specimens. We analyzed 44 different cytokines using the Human XL Cytokine Magnetic Luminex assay (RD Biosystems, Minneapolis, MN, USA) according to manufacturer’s protocols.

The primary analysis was mortality through 18 weeks, which accounts for 95% of 5-year mortality in cryptococcal meningitis [13]. We categorized participants by tertile of CSF culture quantification, resulting in four groups: (1) persons with sterile cultures (Sterile), (2) persons with 1–14,700 CFU/mL (Low Tertile), persons with 14,701–206,000 CFU/mL (Middle Tertile), and persons with >206,000 CFU/mL (High Tertile). We compared baseline characteristics across groups by Chi-square for categorical variables or Kruskall-Wallis for continuous variables. Cytokines were analyzed by comparing the log2 mean of any given biomarker across all groups using unadjusted linear regression. We prioritized identifying biomarkers that met criteria for both *p*-value <0.05 and that were at least 2-fold different in the sterile group versus any of the fungal growth tertiles. Both 30-day mortality and 18-week mortality were compared by Chi-square testing of group proportions. We used a Kaplan–Meier time-to-event model with log-rank testing to visualize 18-week mortality by each group, and a Cox proportional hazards model to determine hazard ratios by tertile grouping. Survival analyses presented in the main manuscript are unadjusted. As a sensitivity analysis, we included two models adjusting for the impact of CD4 count and receiving ART. The first model adds baseline CD4 count as a co-variate. The second model adds both baseline CD4 count and baseline ART receipt as co-variates. Both models account for missing values using a multivariate imputation by chained equations (MICE) methodology. Analyses were completed using both SAS (SAS Institute; Cary, NC, USA) and R (R Foundation; Vienna, Austria).

All participants provided written informed consent as part of the parent trials, including consent for the future testing of clinical samples for research purposes. IRB approval was obtained from the appropriate Ugandan and US institutions.

## 3. Results

We enrolled a total of 765 HIV-positive participants with cryptococcal meningitis for this analysis. Of those, 55 (7%) participants had sterile CSF cultures. By CSF quantitative tertile, 235 participants were grouped in the low 1–14,700 CFU/mL category, 234 participants in the middle 14,701–206,000 category, and 241 participants in the high >206,000 CFU/mL category. Compared across the tertile groupings, those with sterile cultures had significantly higher CD4 counts (median 70 cells/µL [IQR, 25–96]) and were more likely to be receiving ART at time of diagnosis (71% [39/55]) (Table 1). The sterile culture group also had significantly fewer participants with lumbar puncture opening pressures >250 mm H_2_O (21% [10/55]) and lower overall mean opening pressure (170 mm H_2_O [11,12,13,14,15,16,17,18,19,20,21,22,23,24]). Finally, the proportion with CSF pleocytosis (>5 white cells/µL) was significantly higher in both the sterile group (46% [23/55]) and low tertile group (54% [124/235]) compared with the high tertile group (26% [61/241]).

**Figure 1:**
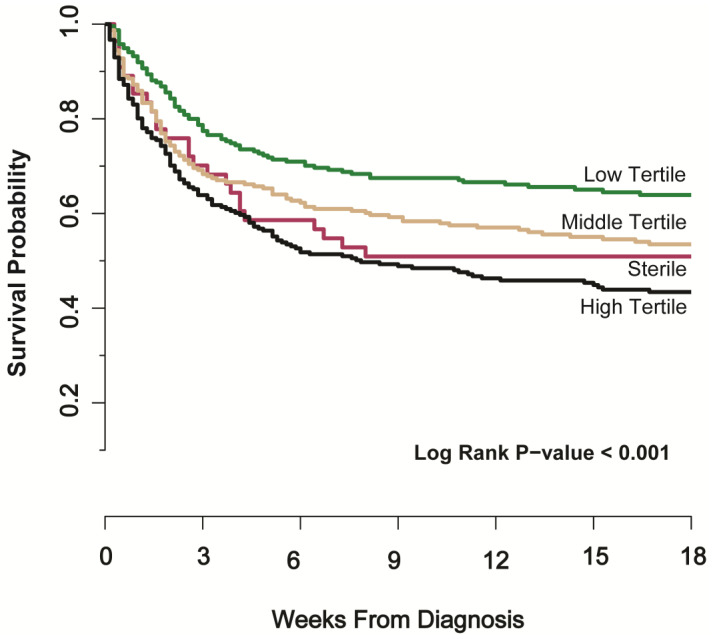
Kaplan–Meier Curve of 18-week Survival by CSF Fungal Burden Tertile in Cryptococcal Meningitis. Figure 1 demonstrates cumulative survival probability by Kaplan–Meier curve with Log Rank testing. At 18 weeks, the high tertile group has the poorest survival with 56% [135/241] dead, followed by the sterile culture group with 47% [26/55] dead, the middle tertile group with 46% [107/234] dead, and the low tertile group with 35% [83/235] dead. Log Rank *p*-value is calculated across all groups. CSF quantitative culture tertiles: Low Tertile = 1–14,700 CFU/mL; Middle Tertile = 14,701–206,000 CFU/mL; High Tertile > 206,000 CFU/mL.

We analyzed mortality by CSF quantitative culture tertile. By 30 days, 40% [22/55] died in the sterile culture group versus 26% [62/235] in low tertile, 34% [79/234] in the middle tertile, and 41% [98/241] in the high tertile groups (*p* < 0.01). Similarly through 18 weeks, 47% [26/55] died in the sterile culture group versus 35% [83/235] in low tertile, 46% [107/234] in the middle tertile, and 56% [135/241] in the high tertile groups (*p* < 0.001) (Figure 1). When analyzing survival through 18 weeks in a time-to-event unadjusted Cox regression model (using the low tertile group as reference), the sterile group had a mortality hazard ratio of 1.57 [95%CI, 1.01–2.44], falling between the middle tertile group (hazard ratio = 1.42 [95%CI, 1.07–1.89]) and the high tertile group (hazard ratio = 1.88 [95%CI, 1.43–2.48]) in terms of risk. As a sensitivity analysis, we created two multivariate models to adjust for CD4 count and ART receipt (Table 2). The hazard ratio for the sterile group was similar (hazard ratio = 1.55 [95%CI, 0.99–2.45]) after adjustment for CD4 count and ART status.

Persons with sterile cultures were more likely to be receiving ART than persons in the other tertiles. Given the high mortality found in the sterile culture group, we analyzed the timing of ART initiation in this group. We found that persons with sterile cultures initiated ART a median of 2.3 months (IQR, 1.1–12.8) prior to diagnosis, a shorter time frame as compared to the low tertile group at 4.1 months (0.8–19.7), although this difference was not statistically significant (Table 1).

A subset of 334 participants had baseline CSF cytokine measurements, including 24 persons in the sterile group. Of the 44 cytokines quantified from CSF in our participants, a total of 7 were found in statistically higher concentrations in the sterile group compared to those with any tertile of fungal growth (Table 3). The cytokines found to be at least 2-fold higher in the sterile group include: interleukin (IL)-6, IL-17, interferon (IFN)-γ, IFN-α, granulocyte colony-stimulating factor (G-CSF), granulocyte-macrophage colony-stimulating factor (GM-CSF), and chemokine CXCL2 (macrophage inflammatory protein 2-alpha). No cytokines measured were two-fold lower in the sterile culture group. A full table of all 44 cytokines categorized by functional group can be found in Supplemental Table S1.

## 4. Discussion

Overall, our results demonstrate that persons with HIV-associated cryptococcal meningitis but without fungal growth in CSF have higher early 30-day mortality, comparable to those with the highest fungal burden. By 18 weeks, mortality falls between the middle and high tertile groups, but remains significantly higher than the low tertile group. The traditional paradigm considered increasing CSF fungal burden as having increased risk of mortality. However, with the introduction of highly sensitive, non-culture based diagnostics [4], cases of cryptococcal meningitis without fungal growth are increasingly recognized [9]. Until now, it was not known how cryptococcal meningitis outcomes might differ between those with sterile cultures versus those with varying degrees of fungal burden. We have observed that those with sterile cultures have unexpected relatively high mortality, although the cause of that higher mortality is not entirely clear.

We note some surprising features about this cohort. The first being that the sterile culture group paradoxically features several characteristics classically associated with lower mortality in cryptococcal meningitis. Only 21% [10/55] of the sterile culture group had substantially elevated CSF opening pressures (>250 mm H_2_O), compared to all other tertiles having at least 45% of persons with high opening pressures. Prior studies have demonstrated that high baseline opening pressures may be associated with increased mortality [14,15], and control of intracranial pressure is associated with improved survival in cryptococcal meningitis [16]. Thus, one could expect that the sterile group might have better outcomes given that significantly fewer participants in this group have elevated opening pressures. Second, persons with sterile cultures had higher levels of CSF white cells than those with the highest fungal burden, suggesting a more robust immune response in the central nervous system. Lack of white blood cells in the CSF has previously been linked to poorer outcomes [5], and we otherwise find a fairly linear relationship of decreasing CSF white cells with increasing fungal burden in those with CSF growth. Thus, it was surprising to find increased CSF cellular infiltrate in our high mortality sterile group, which lead us to additional inquiries about the immune response in persons with sterile CSF.

In order to better understand the immune response in the sterile culture group, we compared quantitative CSF cytokine measurements across the groups. We found seven cytokines to be significantly higher in the sterile culture group as compared to any fungal growth tertile: IL-6, IL-17, IFN-γ, IFN-α, G-CSF, GM-CSF, and CXCL2. To date, IFN-γ, IL-6, G-CSF, and GM-CSF have been the best described in the literature. A principal component analysis by Jarvis et al. found that IL-6 and IFN-γ were key factors associated with rapid clearance of Cryptococcus yeast from the CSF [17]. Interestingly, Jarvis et al. found that IL-17 positively correlated with IFN-γ concentrations and may also play a role in fungal clearance. Herein, we found decreasing IL-17 and decreasing IFN-γ associated with higher fungal burdens. Elevated IFN-γ and G-CSF concentrations have previously been reported in persons who develop paradoxical immune reconstitution inflammatory syndrome (IRIS) [18]. Elevated GM-CSF at diagnosis has been associated with an increased likelihood of later developing paradoxical IRIS [17]. IFN-α is a Type 1 interferon classically associated with upregulation as a viral response, while CXCL2 (MIP2-α) is secreted by macrophages as a chemotactic for polymorphonuclear leukocytes. Neither has been particularly implicated with the cryptococcal disease before. In summary, our participants with sterile CSF cultures have a cytokine profile that is consistent with fungal clearance, but which may also predispose to an IRIS-like phenomena. However, the elevated concentration of cytokines such as IFN-α and CXCL2 and the lack of other cytokines classically associated with fungal clearance (i.e., TNF-α) [19], in the setting of high mortality, may suggest this is a unique immunologic entity that is not appropriately considered by current treatment guidelines which draw on historically ART-naïve populations.

One hypothesis for the increased cellular infiltrate and upregulated cytokine production seen in the sterile culture group relates to that of immune reconstitution. Persons with sterile cultures were more likely to be receiving ART at the time of their meningitis diagnosis, were more likely to have recently initiated ART, and had significantly higher median CD4 counts. Presumably, more functioning CD4 cells allows for a more robust immune response against the opportunistic infection. However, this may be a proverbial “double-edged sword” in the population. Scriven et al. demonstrated in a randomized trial that persons who started ART early after cryptococcal meningitis diagnosis (and whom subsequently had worse outcome) had increased cellular infiltrate and type 2 T helper responses in the CSF [20]. Additionally, Rhein et al. found that persons with advanced HIV who recently initiated ART and were subsequently diagnosed with meningitis within 2 weeks had poorer outcomes than those who were ART-naïve [7]. Termed “unmasking” disease, the hypothesis is that ART-triggered recovery of CD4 cells and reactivation of the immune system may be detrimental in persons with cryptococcal infection without first treating with antifungal therapy [21,22].

**Figure 2:**
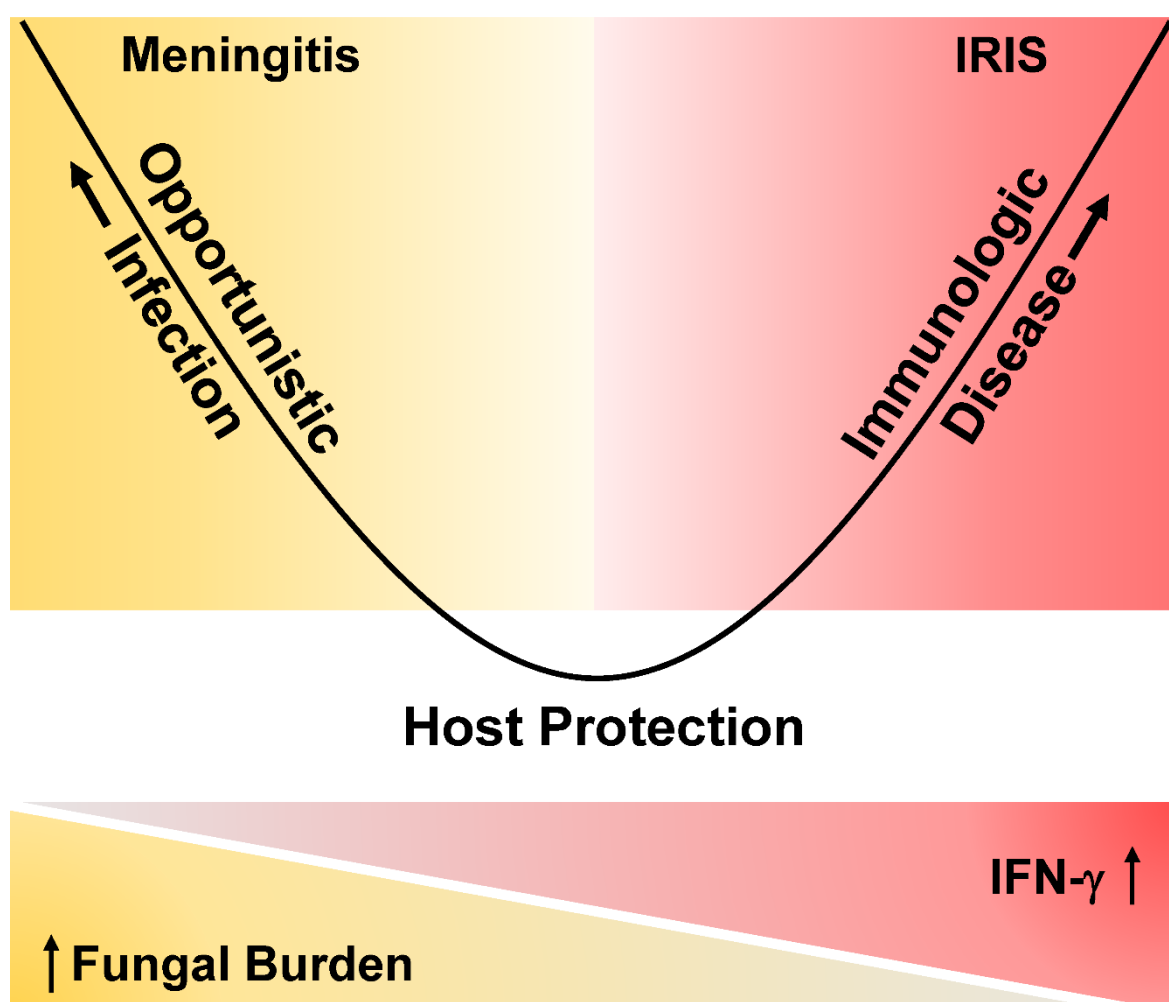
Simplified Damage-Response Parabolic Framework Model of Cryptococcal Meningitis Disease. Figure 2 demonstrates a hypothesized damage-response model for HIV-associated cryptococcal disease. The model proposes that clinical disease can occur by either of two mechanisms: (1) Uncontrolled fungal dissemination characterized by suboptimal IFN-γ predominated Type 1 helper (Th_1_) response with increasing CSF quantitative culture burden, or (2) Excessive damaging host immune response characterized by exaggerated IFN-γ predominated Th_1_ host response with persons presenting with sterile CSF cultures. The host is most likely protected by a moderate, balanced immune response. (Figure is originally adapted from Pirofski and Casadevall concept, and further modified from our prior publication [29]).

Pirofski and Casadevall proposed the damage-response framework model whereby clinical disease can occur by either uncontrolled pathogen dissemination or a detrimental host immune response, but with the common denominator being host damage (Figure 2) [23,24]. Immune reconstitution inflammatory syndrome (IRIS) is a dysregulated immune response to an opportunistic pathogen upon restoration of a depleted component of the immune system [22]. Excess inflammation in the brain in cryptococcal meningitis IRIS can be fatal [25,26]. In contrast to unmasking disease, paradoxical cryptococcal IRIS occurs subsequent to diagnosis and treatment of infection and after initiating ART. Paradoxical IRIS may present anywhere from a few days to years after ART initiation in cryptococcal meningitis, while the incidence and mortality are variable, with incidence ranging between 8–49% and mortality between 0–36% [22,27,28]. While unmasking cryptococcal disease and paradoxical cryptococcal IRIS occur at different time points in relation to diagnosis, the pathogenesis of the two may be similar.

Alternatively, the unique pathogenesis of this sterile cohort may not be due to ART-associated unmasking IRIS, but rather some other immune related entity. In contrast to the Rhein et al. findings, the larger ACTA trial group observed only weak evidence of a trend toward increased mortality in those recently initiating ART, reporting a 2-week mortality of 23% for those initiating ART within 2 weeks prior to diagnosis compared to 15% initiating ART >2 weeks (Hazard Ratio = 1.70; 95%CI, 0.85–3.39; *p* = 0.13) [30]. One possibility is that the specific strain of *Cryptococcus* may be driving the unique immune response. Nielsen and colleagues have demonstrated in mouse models that different clinically isolated human *Cryptococcus* strains yield different survival outcomes in the mice, and have identified numerous genes that may influence this differential pathogenicity [31,32]. Unfortunately, identifying strain specific disease in real time is not currently possible, but would certainly be a future area of research interest. Lastly, we feel misidentified disease due to false positive antigen testing is highly unlikely given the clinical presentation is consistent with cryptococcal meningitis, the fact that the majority do still respond to treatment, and the well-established excellent specificity of the CrAg LFA [4].

A limitation in studying the global cytokine profile of a severely immunocompromised person is the potential for other pathogens to influence the findings. All of our patients were admitted for presentations consistent with meningitis but may have had other opportunistic infections. In particular, the presence of IFN-α may suggest the presence of viral co-infections in higher proportions in the sterile culture group. Unfortunately, we do not consistently collect HIV viral loads in this cohort, which could also influence IFN-α. However, our large sample size of similarly immunocompromised persons should reflect a relatively even distribution of concomitant opportunistic infections. We cannot determine how cytokine profiles might have changed over time as our measurements are at baseline. We do not have paired blood cytokine measurements to determine if the CSF findings are a compartmentalized phenomenon versus a whole body response. It is possible that in rare situations cryptococcal yeast might not grow within 10 days of culture, and it is therefore possible persons in our cohort considered sterile might have had growth after 10 days. Lastly, we cannot generalize these findings to the subset of patients who have HIV-negative cryptococcal meningitis; however these findings may provide insight into the diversity of immune responses present in HIV-negative meningitis.

The other major limitation of our study was our survival analysis. We recognize that there are limitations with either an unadjusted or partially adjusted survival analysis, but we felt it valuable to draw early attention to the surprising features of this group to facilitate future study. We performed a sensitivity analysis to determine if the hazard ratios were similar when adjusting for baseline CD4 count and ART receipt. In general, the hazard ratio for the sterile culture group is similar (Table 2). We do think there are several features that are reassuring that an association exists. One, the hazard ratio measures the effect size, which is maintained after adjustment; if sample size was increased, we would anticipate the effect size would be similar but the power to detect a difference would increase. Two, neither CD4 count or ART receipt were independently associated with mortality. Additionally, three, we would argue that receiving ART at baseline (and subsequently higher CD4 counts) are in the causal pathway for CSF culture sterility in persons with cryptococcal meningitis. Variables in the causal pathway are not considered confounders and not typically adjusted for. The scope of this manuscript was to highlight the high level differences in this sterile culture group. Ultimately, a robust study would need to explore the complex interactions of ART status, adherence, timing of initiation, and immune reconstitution timing and quality to better understand these potentially complex interactions.

In conclusion, we find that persons with sterile CSF cultures at cryptococcal meningitis diagnosis have high mortality, similar to those with higher fungal burdens. This finding is particularly unexpected given the higher CD4 cell count, lower CSF opening pressures, and IFN-γ predominant CSF pro-inflammatory cytokine profile of the group. Our observations challenge the traditional paradigm that increasing CSF fungal burdens are associated with increased mortality. However, sterile culture cryptococcal meningitis may rather represent a unique disease entity consistent with a parabolic damage-response framework model. Further research is needed from a prospective, controlled cohort.

## Figures and Tables

**Table 1:** Baseline characteristics by CSF quantitative culture burden in first-episode HIV-related cryptococcal meningitis.

CSF Fungal Burden	Sterile	Low CFU Tertile *	Middle CFU Tertile *	High CFU Tertile *	*p*-Value
	(N = 55)	(N = 235)	(N = 234)	(N = 241)	
**Demographics**					
Age, years	37 [30, 42]	34 [29, 40]	36 [30, 40]	35 [29, 40]	0.09
Women	27 (49.1%)	99 (42.1%)	84 (35.9%)	100 (41.5%)	0.25
Weight, kg	55 [50, 60]	53 [47, 60]	52 [47, 60]	52 [48, 59]	0.52
Glasgow Coma Score < 15	25 (45.5%)	98 (41.9%)	81 (34.8%)	110 (45.6%)	0.09
CD4 Count, cells/μL	70 [25, 96]	25 [9, 62]	15 [6, 39]	10 [6, 28]	<0.001
Receiving HIV Therapy	39 (70.9%)	108 (46.2%)	75 (32.1%)	63 (26.1%)	<0.001
Months of HIV Therapy †	2.3 [1.1, 12.8]	4.1 [0.8, 19.7]	3.0 [0.7, 24.4]	15.0 [1.1, 38.8]	0.10
**Baseline CSF Parameters**					
Opening Pressure > 250 mm H_2_O	10 (20.8%)	89 (45.2%)	125 (60.7%)	145 (67.1%)	<0.001
Opening Pressure, mm H_2_O	168 [113, 240]	230 [160, 340]	288 [200, 401]	348 [220, 473]	<0.001
CSF > 5 White Cells/μL	23 (46.0%)	124 (54.1%)	72 (32.3%)	61 (26.2%)	<0.001
CSF Protein, mg/dL	55 [20, 124]	76 [28, 140]	57 [23, 97]	56 [24, 117]	0.13

Data are N (%) or medians with [P25, 75]. *p*-value from Kruskal–Wallis test for medians, Chi-squared test for proportions. * Fungal burden tertiles: Low tertile = 1–14,700 CFU/mL; Middle tertile = 14,701–206,000 CFU/mL; High tertile > 206,000 CFU/mL. † Among those on ART at diagnosis. Abbreviations: CFU = colony forming units; CSF = cerebrospinal fluid. Data missingness as follows: Age (N = 0), Sex (N = 0), Weight (N = 165), Glasgow Coma Score (N = 2), CD4 (N = 40), Receiving HIV Therapy (N = 0), Months of HIV Therapy (N = 195), Opening pressure (N = 98), CSF White Cells (N = 30), CSF Protein (N = 96).

**Table 2:** Proportional Hazards Regression Models for 18 Week Mortality by CSF Quantitative Culture Burden using Low Tertile Grouping (1–14,700 CFU/mL) as the Reference Group.

Variable	Hazard Ratio	Lower Bound 95%CI	Upper Bound 95%CI
Univariate Unadjusted [N = 765]			
Sterile Group	1.57	1.01	2.44
Middle Tertile, 14,701–206,000 CFU/mL	1.42	1.07	1.89
High Tertile, >206,000 CFU/mL	1.88	1.43	2.48
Multivariate Adjusted Model #1 [N = 765]			
Sterile Group	1.63	1.04	2.56
Middle Tertile, 14,701–206,000 CFU/mL	1.40	1.05	1.87
High Tertile, >206,000 CFU/mL	1.84	1.40	2.45
CD4^+^ T cell count, per 10 cells/μL	1.00	0.99	1.01
Multivariate Adjusted Model #2 [N = 765]			
Sterile Group	1.55	0.99	2.45
Middle Tertile, 14,701–206,000 CFU/mL	1.43	1.07	1.91
High Tertile, >206,000 CFU/mL	1.90	1.44	2.52
CD4^+^ T cell count, per 10 cells/μL	1.00	0.99	1.01
Receiving ART at baseline	1.17	0.94	1.47

ART = antiretroviral therapy. Missing values for CD4 (N = 40) were imputed using the multivariate imputation by chained equations (MICE) method (in R). Interpretation: We performed a sensitivity analysis to assure the hazard ratios were similar when adjusting for baseline CD4 count and ART receipt. In the first model, CD4 is added as a continuous co-variate. In the second model, CD4 is added as a continuous co-variate and receiving ART at baseline is added as a categorical co-variate. The hazard ratio for the sterile culture group is very similar. We do note that after adjustment in model #2, the 95%CI for the sterile culture group “crosses one” by less than one-hundreth of a point. Despite the fact that would yield a *p*-value slightly greater than 0.05, we feel strongly that an association likely exists for the following reasons. One, the hazard ratio measures the effect size, which is maintained after adjustment; if sample size was increased, we would anticipate the effect size would be similar but the power to detect a difference would increase. Two, neither CD4 count (hazard ratio = 1.00 [95%CI, 0.99–1.01]) or ART receipt (hazard ratio = 1.17 [95%CI, 0.94–1.47]) was independently associated with mortality. Additionally, three, we would argue that receiving ART at baseline (and subsequently higher CD4 counts) are in the causal pathway for CSF culture sterility in persons with cryptococcal meningitis. Variables in the causal pathway are not considered confounders and are typically not adjusted for. We further examine this concept in our damage-response framework model in the discussion.

**Table 3:** CSF biomarkers with more than two-fold difference between those with sterile cultures and those with fungal growth as quantified on baseline CSF.

	Sterile CSF (N = 24)	Low CFU Tertile (N = 98)	Middle CFU Tertile (N = 99)	High CFU Tertile (N = 113)	*p*-Value
LABS	Median [IQR]				
CD4 cells/μL	64 [26, 121]	20 [7, 49]	15 [6, 32]	10 [6, 30]	<0.001
CSF white cells/μL	25 [4, 155]	20 [<5, 95]	<5 [<5, <5]	<5 [<5, <5]	<0.001
CYTOKINES	Mean (95% CI) on log_2_ pg/mL scale				
IL-6	9.2 (7.8, 10.6)	8.3 (7.6, 8.9)	7.4 (6.7, 8.1)	7.4 (6.9, 8.0)	0.02
IL-17	2.0 (0.8, 3.1)	0.6 (0.0, 1.2)	−0.2 (−0.7, 0.4)	−0.3 (−0.8, 0.2)	<0.001
IFN-γ	3.0 (2.0, 3.9)	1.7 (1.2, 2.2)	1.4 (0.9, 1.8)	1.3 (0.9, 1.7)	0.02
G-CSF	7.0 (5.9, 8.1)	5.1 (4.6, 5.6)	4.7 (4.3, 5.0)	5.0 (4.6, 5.3)	<0.001
GM-CSF	6.2 (5.4, 7.0)	5.2 (4.9, 5.5)	4.9 (4.6, 5.2)	5.0 (4.7, 5.2)	<0.001
CXCL2 (GRO-b)	4.8 (3.9, 5.6)	3.5 (3.0, 3.9)	3.3 (2.8, 3.7)	3.7 (3.3, 4.0)	0.02
IFN-α	3.1 (2.2, 3.9)	1.6 (1.0, 2.2)	1.7 (1.3, 2.1)	2.1 (1.7, 2.4)	0.02

CSF quantitative culture tertiles: Low Tertile = 1–14,700 CFU/mL; Middle Tertile = 14,701–206,000 CFU/mL; High Tertile > 206,000 CFU/mL. *p*-value from unadjusted linear regression. Abbreviations: IQR, interquartile range; 95% CI, 95% confidence interval; CSF, cerebrospinal fluid; CFU, colony forming units; IL-6, interleukin 6; IL-17, interleukin 17; IFN-γ, interferon gamma; G-CSF, granulocyte colony stimulating factor; GM-CSF, granulocyte macrophage colony stimulating factor; CXCL2 (GRO-b), growth regulated protein beta; IFN-α, interferon alpha. Full set of CSF biomarkers provided as Supplemental Table S1.

## Data Availability

The data presented in this study are available on request from the corresponding author in a de-identified dataset.

## Supplementary Materials

The following supporting information can be downloaded at: https://www.mdpi.com/article/10.3390/jof9010046/s1, Supplemental Table S1. Baseline Mean CSF Biomarkers by CSF Quantitative Culture Tertile.
