# Supplementary material for: Sterile Cerebrospinal Fluid Culture at Cryptococcal Meningitis Diagnosis Is Associated with High Mortality
Source: J Fungi (Basel). 2022 Dec 28;9(1):46. doi: 10.3390/jof9010046 (PMC9866844; doi:10.3390/jof9010046)
Supplement: Supplementary file 1 [file jof-09-00046-s001.zip › jof-2012976-supplementary.pdf]

Supplemental Table 1: Baseline Mean CSF Biomarkers by CSF Quantitative Culture Tertile

Biomarkers log_2 pg/mL	CSF Culture Sterile		CSF CFU 1-14,700		CSF CFU 14,701-206,000		CSF CFU >206,000		P-value
	N	Mean (95% CI)	N	Mean (95% CI)	N	Mean (95% CI)	N	Mean (95% CI)	
**Mediate inflammatory responses**									
IL-1α	24	2.0 (1.1, 2.8)	98	1.0 (0.5, 1.5)	99	1.3 (0.9, 1.7)	113	1.3 (1.0, 1.7)	0.23
IL-6	24	9.2 (7.8, 10.6)	98	8.3 (7.6, 8.9)	99	7.4 (6.7, 8.1)	113	7.4 (6.9, 8.0)	0.02
IL-1β	24	1.9 (1.0, 2.8)	98	0.7 (0.2, 1.2)	99	0.9 (0.6, 1.3)	113	1.0 (0.6, 1.3)	0.09
PDGF-aa	24	6.9 (6.4, 7.4)	98	6.2 (5.9, 6.5)	99	6.2 (6.0, 6.4)	113	6.3 (6.1, 6.6)	0.08
PDGF-ab	24	1.0 (-0.3, 2.3)	98	-0.2 (-0.8, 0.4)	99	-0.1 (-0.8, 0.6)	113	-0.2 (-0.9, 0.4)	0.39
VEGF	24	6.1 (5.7, 6.6)	98	5.5 (5.2, 5.8)	99	5.3 (5.0, 5.5)	113	5.5 (5.3, 5.8)	0.05
FGF-basic	24	0.2 (-0.8, 1.2)	98	0.0 (-0.4, 0.4)	99	0.2 (-0.2, 0.6)	113	-0.3 (-0.7, 0.1)	0.31
**T-helper cell Type 1 (Th_1) immune response**									
IL-12	24	2.6 (1.8, 3.4)	98	1.6 (1.1, 2.1)	99	1.9 (1.6, 2.3)	113	2.1 (1.8, 2.4)	0.12
IL-15	24	1.5 (1.1, 2.0)	98	1.1 (0.7, 1.6)	99	1.5 (1.2, 1.8)	113	1.7 (1.4, 1.9)	0.13
IFN-γ	24	3.0 (2.0, 3.9)	98	1.7 (1.2, 2.2)	99	1.4 (0.9, 1.8)	113	1.3 (0.9, 1.7)	0.02
TNF-α	24	5.6 (4.7, 6.5)	98	5.0 (4.5, 5.5)	99	5.1 (4.7, 5.6)	113	5.2 (4.9, 5.6)	0.70
GM-CSF	24	6.2 (5.4, 7.0)	98	5.2 (4.9, 5.5)	99	4.9 (4.6, 5.2)	113	5.0 (4.7, 5.2)	<0.001
GranzymeB	24	4.5 (3.3, 5.7)	98	3.5 (2.9, 4.1)	99	3.4 (2.9, 3.9)	113	3.4 (2.9, 3.8)	0.30
TRAIL	24	3.2 (2.4, 4.0)	98	2.0 (1.5, 2.5)	99	2.1 (1.6, 2.6)	113	2.2 (1.9, 2.6)	0.14
CCL2 (MCP1)	24	9.6 (9.2, 10.1)	98	9.6 (9.4, 9.9)	99	9.8 (9.5, 10.1)	113	10.2 (9.9, 10.4)	0.03
CCL3 (MIP1α)	24	5.1 (4.4, 5.8)	98	4.6 (4.2, 5.0)	99	5.2 (4.9, 5.5)	113	5.4 (5.2, 5.6)	<0.01
CCL4 (MIP1β)	24	7.5 (6.9, 8.2)	98	7.1 (6.8, 7.4)	99	7.3 (7.1, 7.5)	113	7.3 (7.1, 7.5)	0.53
CCL5 (RANTES)	17	5.8 (4.5, 7.1)	66	4.7 (4.0, 5.3)	65	4.8 (4.2, 5.5)	78	4.5 (3.9, 5.1)	0.35
CXCL10 (IP10)	24	10.9 (10.2, 11.5)	98	10.9 (10.6, 11.2)	99	10.6 (10.3, 10.9)	113	10.3 (9.9, 10.7)	0.09
**T-helper cell Type 2 (Th_2) immune response**									
IL-4	24	-0.3 (-1.0, 0.4)	98	-1.1 (-1.7, -0.4)	99	-0.1 (-0.6, 0.3)	113	-0.2 (-0.7, 0.3)	0.08
IL-5	24	0.8 (-0.3, 1.9)	98	-0.2 (-0.8, 0.3)	99	-0.5 (-1.0, 0.0)	113	-0.7 (-1.1, -0.3)	0.07
IL-13	24	4.4 (3.7, 5.0)	98	3.8 (3.5, 4.2)	99	4.0 (3.6, 4.3)	113	4.0 (3.7, 4.3)	0.51
IL-33	24	1.9 (0.3, 3.5)	98	1.4 (0.7, 2.1)	99	1.3 (0.5, 2.1)	113	1.2 (0.5, 2.0)	0.89
IL-25	24	3.3 (2.5, 4.0)	98	2.7 (2.1, 3.3)	99	3.1 (2.6, 3.7)	113	3.4 (2.9, 3.8)	0.31
Eotaxin	24	3.7 (3.0, 4.4)	98	3.1 (2.8, 3.5)	99	3.2 (2.9, 3.6)	113	3.3 (3.0, 3.6)	0.52
**Type 3 (Th_{17}) immune response**									
IL-17	24	2.0 (0.8, 3.1)	98	0.6 (0.0, 1.2)	99	-0.2 (-0.7, 0.4)	113	-0.3 (-0.8, 0.2)	<0.01
G-CSF	24	7.0 (5.9, 8.1)	98	5.1 (4.6, 5.6)	99	4.7 (4.3, 5.0)	113	5.0 (4.6, 5.3)	<0.001
CXCL1 (GROA)	24	8.3 (7.3, 9.3)	98	7.1 (6.5, 7.7)	99	7.1 (6.6, 7.6)	113	7.1 (6.7, 7.5)	0.22
CXCL2 (GROB)	24	4.8 (3.9, 5.6)	98	3.5 (3.0, 3.9)	99	3.3 (2.8, 3.7)	113	3.7 (3.3, 4.0)	0.02
IL-8 (CXCL8)	24	9.4 (8.5, 10.4)	98	8.7 (8.2, 9.2)	99	8.6 (8.2, 9.1)	113	8.7 (8.3, 9.1)	0.51
CCL20 (MIP3α)	24	2.7 (2.0, 3.4)	98	1.8 (1.4, 2.2)	99	1.8 (1.5, 2.2)	113	1.8 (1.5, 2.2)	0.17

**Modulate/dampen Immune Responses**									
IL-1ra	24	11.2 (10.2, 12.3)	98	11.5 (11.1, 11.9)	99	11.7 (11.3, 12.0)	113	12.0 (11.7, 12.3)	0.14
IL-10	24	7.9 (7.1, 8.6)	98	7.8 (7.6, 8.1)	99	7.7 (7.5, 7.9)	113	7.6 (7.4, 7.9)	0.69
IL-2	24	2.6 (2.0, 3.2)	98	1.9 (1.4, 2.3)	99	2.1 (1.8, 2.4)	113	2.0 (1.7, 2.3)	0.29
TGF-α	24	3.0 (2.2, 3.8)	98	2.5 (2.1, 3.0)	99	2.8 (2.5, 3.1)	113	3.0 (2.6, 3.3)	0.42
EGF	24	0.4 (-0.8, 1.6)	98	0.2 (-0.3, 0.7)	99	0.3 (-0.2, 0.8)	113	0.4 (-0.1, 1.0)	0.92
**Immune Exhaustion**									
PD-L1	24	7.1 (6.5, 7.7)	98	6.4 (6.0, 6.8)	99	6.4 (6.1, 6.7)	113	6.5 (6.2, 6.8)	0.34
**Other**									
CCL19 (MIP3β)	24	7.4 (6.4, 8.4)	98	6.7 (6.3, 7.1)	99	6.3 (5.9, 6.6)	113	6.0 (5.7, 6.3)	<0.01
TNFSF5 (CD40L)	24	8.5 (7.6, 9.4)	98	7.8 (7.3, 8.3)	99	7.8 (7.4, 8.3)	113	7.9 (7.6, 8.3)	0.53
Fractalkine (CS3CL1)	24	8.7 (8.3, 9.0)	98	8.0 (7.6, 8.3)	99	7.9 (7.6, 8.2)	113	8.1 (7.9, 8.4)	0.20
Flt3Ligand	24	7.1 (6.8, 7.5)	98	6.5 (6.2, 6.7)	99	6.5 (6.3, 6.7)	113	6.5 (6.3, 6.7)	0.05
IFN-α	24	3.1 (2.2, 3.9)	98	1.6 (1.0, 2.2)	99	1.7 (1.3, 2.1)	113	2.1 (1.7, 2.4)	0.02
IFN-β	24	-1.0 (-2.1, 0.1)	98	-1.3 (-1.9, -0.8)	98	-1.0 (-1.5, -0.6)	113	-1.1 (-1.6, -0.6)	0.83
IL-3	24	0.9 (-0.3, 2.2)	98	1.1 (0.6, 1.7)	99	1.3 (0.7, 2.0)	113	1.4 (0.8, 2.0)	0.87
IL7	24	0.9 (0.1, 1.7)	98	0.6 (0.2, 1.0)	99	0.7 (0.3, 1.1)	113	0.8 (0.5, 1.1)	0.78

Values presented as log2 pg/mL. P-value from unadjusted linear regression.
